# Sustainability in Telehomecare: Exploring Practical Nurses’ Perceptions, Engagement, and Leadership Implications

**DOI:** 10.1155/jonm/5772385

**Published:** 2026-03-12

**Authors:** Melanie Rydgren, Anna Anåker, Linda Estman, Malin Andtfolk, Lisbeth Fagerström

**Affiliations:** ^1^ Department of Natural and Health Sciences, Åbo Akademi University, Vaasa, Finland, abo.fi; ^2^ School of Health and Welfare, Dalarna University, Falun, Sweden, du.se; ^3^ Department of Caring and Ethics, University of Stavanger, Stavanger, Norway, uis.no

## Abstract

**Background:**

Healthcare systems are adapting to environmental challenges and digital transformations. Practical nurses’ perceptions and engagement are key to advancing sustainability in everyday care in a digital setting. Hence, it is essential to explore whether practical nurses within telehomecare settings are prepared for the adoption of sustainable practices and the implications of their perceptions of sustainability within nursing practice for nursing leadership.

**Aim:**

To explore how practical nurses perceive and engage with sustainability within telehomecare.

**Design:**

This study had a qualitative exploratory design.

**Methods:**

Ten practical nurses working in telehomecare were interviewed between February and April 2024 in Finland. The interviews were individual and semistructured. Data were analyzed through reflexive thematic analysis.

**Results:**

The main themes identified in this study, *Emerging Eco-Consciousness in Telehomecare* and *Constrained Eco-Engagement in Telehomecare,* encapsulate the tension between emerging environmental awareness and the limitations of telehomecare settings. The participants demonstrated limited awareness of and engagement with sustainability in their professional roles. Moreover, they regarded the concept of sustainability as ambiguous and had a fragmented understanding of what it entails in the context of healthcare. Their capacity to actively engage in sustainable practices was shaped by challenges related to assuming responsibility, the need to balance professional values with the potential negative outcomes of sustainable digital practices, and limited opportunities for involvement in key organizational decision‐making processes.

**Conclusion:**

This study highlights a need to cultivate greater consciousness among healthcare professionals and leaders of the critical role of sustainability in healthcare delivery. The results highlight the importance of integrating targeted education and training on environmental issues and sustainable practices to foster climate–health literacy and deepen engagement and of equipping leaders to champion green transitions. Enhancing knowledge and competence in sustainability can, in combination with an ethical, green, transformational leadership style, support organizational shifts toward more environmentally sustainable healthcare systems.

## 1. Introduction

Alongside the ongoing digital transformation of healthcare services, more attention is being directed to implementing more sustainable practices in nursing. The healthcare sector is recognized as a contributor to climate change, with processes that add to greenhouse gas emissions and require an extensive use of climate resources [1]. The concept of sustainability in healthcare encompasses environmental, economic, and social dimensions [2]. Sustainable healthcare aims to deliver high‐quality care while minimizing the sector’s environmental footprint and ensuring equitable access to services [3]. From a nursing perspective, means to achieve this aim can include sustainability integration, environmental advocacy, holistic patient care, professional development and education, collaboration, and leadership [4]. Telenursing services involving remote care consultations [5] have the potential to contribute to more sustainable healthcare delivery [6, 7]. One such service is telehomecare, which involves practical nurses contacting clients living at home through a video‐communication device [5]. Clear proof exists of the advantages of sustainable practices and of their alignment with professional nursing values and ethos [4, 8]. Nonetheless, their implementation in healthcare settings has been challenging [9], which underlines the importance of successful leadership in the changing work environment. Nursing leadership in digital care settings, such as telehomecare, must make strategic efforts to ensure effectiveness, innovation, transformation, and personnel support, with an orientation toward knowledge and competence [10]. Furthermore, nursing leaders, as fosterers of a culture of change [11] and gatekeepers leading the implementation of new technology within their workplace [12], must maintain a strong commitment throughout transformational processes [8], engaging with and motivating nurses to perform this form of transformation [13].

Nurses and other care professionals require knowledge, competencies, and organizational support to be able to make meaningful contributions to development and implementation processes concerning sustainability [14–16]. However, nurses’ *eco-engagement*—whereby the individual actively chooses environmentally friendly practices [17]—may be hindered by a lack of awareness and knowledge about sustainability and climate impact [18, 19] and of practical tools to engage in sustainable methods of working in their clinical practice [8]. Additionally, individual attitudes and the complexity of the setting may influence implementation processes. A time‐sensitive and hectic working environment and the professional core value of putting the client’s needs first [8, 20], as well as secure aseptic working methods to prevent the spread of infections [21], may hinder nurses’ engagement with sustainability. According to multiple studies, some nurses struggle to recognize the connection between climate change, sustainable development, and nursing practices [22, 23], revealing the need for more climate–health literacy among healthcare professionals [24]. While many nurses view sustainability as an ethical responsibility, their ability to act sustainably is often limited by external constraints, such as weak policies and insufficient infrastructure [8, 22], competing priorities and lack of resources [19, 25], unsustainable procurement decisions [20, 26], lack of organizational support and commitment [9, 20, 22, 26], and hierarchical constraints [9, 22].

Despite these challenges, nurses ought to play an active role in shaping sustainability‐related policies within healthcare systems [27]. The nursing profession is at the forefront of managing the health impacts of climate change [28] and has a crucial role in protecting and preserving the environment by adopting sustainable working practices [15] through knowledge, attitudes, and skills [29]. This form of *eco-conscious nursing*, where nurses as individuals wholeheartedly embrace sustainability in their work, can mitigate the climate impact of healthcare and enhance patient outcomes [4]. Nurses’ engagement with and awareness of sustainability in traditional care settings have been explored in previous research to some extent; however, there is a notable lack of research examining the topic specifically within the context of digital healthcare settings [30, 31] and among practical nurses (ISCO‐08 code 3221: nursing associate professional [32]). As digitally mediated nursing services continue to expand, with practical nurses as key healthcare providers, it is vital to understand how practical nurses working in telehomecare perceive and enact sustainability. As frontline providers of care, practical nurses offer perspectives that are essential for a comprehensive understanding of nursing practice, ensuring that strategies are both contextually grounded and operationally feasible. Their insights are essential for identifying the competence requirements and organizational conditions necessary for successfully developing and implementing sustainable digital healthcare practices within the context of everyday nursing work.

A previous study based on the same data collection as the present study focused on how participants perceived telehomecare in relation to sustainability and showed that practical nurses viewed telehomecare as contributing to more sustainable homecare practices [33]. The present study shifts the focus from perceptions of telehomecare to practical nurses’ engagement and consciousness of sustainability within the telehomecare context, as well as the implications for leadership. Eco‐consciousness refers to practical nurses’ awareness, understanding, and attitudes toward sustainability, whereas eco‐engagement captures the extent to which these are translated into practice through behaviors and actions. Therefore, this study explores how practical nurses perceive and engage with sustainability within telehomecare.

## 2. Materials and Methods

### 2.1. Study Design

A qualitative exploratory design was applied in this study [34]. Data collection consisted of holding individual, semistructured interviews with practical nurses. The data were analyzed with an inductive approach through reflexive thematic analysis [35]. The study followed the Standards for Reporting Qualitative Research (SRQR) [36].

The data originate from the same data collection as the qualitative study by Rydgren et al. [33]. However, this article has a separate research focus, is based on a different segment of the material, and applies a distinct analytical lens to explore the study’s aim.

### 2.2. Aim

The study aimed to explore how practical nurses perceive and engage with sustainability within telehomecare.

### 2.3. Study Setting and Participants

In Finland, healthcare, social welfare, and rescue services are organized by regional, self‐governing well‐being services counties. Clients with nonacute conditions can access care services in the home through homecare services [37]. In 2024, it was estimated that 13.1% of people aged over 75 years had regular homecare services in Finland. Furthermore, 5.7% of all homecare services in Finland were provided in digital form, including telephone contact, electronic services, and real‐time remote services [38]. The study was performed in the telehomecare context in three bilingual municipalities in a well‐being services county in Finland. The practical nurses offering telehomecare provide supervisory care where they guide clients in different daily health‐related tasks and assess the clients’ well‐being and medical adherence. The calls are predetermined together with the client, and each session takes approximately 10 min, depending on the needs of the client.

Participants were recruited using purposive sampling within the well‐being services county to ensure they had the experience needed to provide rich, relevant insights from the limited context. The participants were practical nurses with a minimum of 3 months’ experience working with telehomecare. In Finland, practical nurses have completed a 3‐year vocational education program that focuses on primary care practices and provides basic knowledge of health and care [39]. Furthermore, the participating nurses were required to have a minimum of 3 months’ work experience in physical care services for older persons.

The first author obtained the email addresses of the participants from the healthcare leaders in the well‐being services county. Twenty practical nurses working with telehomecare were contacted and asked to participate in the study. Furthermore, the participants were informed of the study through the respective healthcare leaders as well as by direct contact from the first author. Nine practical nurses declined participation, and one of the contacted persons did not meet the inclusion criteria. Information power was assessed using the model proposed by Malterud et al. [40], where the study aim, quality of dialogue, and sample specificity were the most influential determinants. After ten interviews, information power was accomplished based on the data alignment with the study’s aim, the specificity of the sample, and the richness and quality of the interview dialogues.

The ten participants in this study were all female. The median age of the participants was 44.5 years, with age ranging from 28 to 64 years. Four participants were Finnish‐speaking, while six were Swedish‐speaking. The participants had considerable experience in the nursing field, with a median of 20 years of work experience (0.8–42 years). The participants’ experience with telehomecare was more limited, with a median of 0.7 years and a range from 0.4 to 7 years. These participant characteristics show a diverse group of practical nurses who were relatively new to the telehomecare service.

### 2.4. Data Collection

A nine‐question interview guide was developed for this study through author collaboration, consulting leaders and practical nurses in the telehomecare context and examining existing literature on the study subject and methods. Four of the nine interview questions were deemed relevant to the objectives of this study and were therefore included in the analysis, as they aligned with the study aim. During the interviews, the pillars of sustainability within the healthcare context [2] were discussed with each participant to ensure mutual understanding and maintain the research focus. The following questions were used in the interviews conducted for this study: “Can you describe what sustainability in healthcare is and what it means to you? How is sustainability discussed in your workplace/education? How is the climate impact of healthcare discussed in your workplace/education? How have you been involved in the development of the service, and are you able to modify the service?” Some of the remaining questions in the interview guide were used in another study conducted by the same authors [33]. Before data collection, the interview guide received ethical approval from the well‐being services county. As several participants struggled to interpret the topic of sustainability, the interviewer used follow‐up questions and clarified the subject by reformulating the questions and referring to specific examples to facilitate understanding.

All the interviews were performed by the first author, who has significant clinical experience in homecare. The interviews were conducted between February and April 2024 by video, in the participant’s mother tongue (Finnish or Swedish). Before each interview, informed consent was obtained from the participants. The 30–60‐min interviews were video‐ and audio‐recorded. The participants’ nonverbal behaviors, such as expressing discomfort and showing gestures, were noted to support the analysis of the data.

### 2.5. Data Analysis

The first author transcribed the numbered recordings within 5 days of conducting the interviews to maintain data integrity. During the process of transcription, the author listened to the recorded interview multiple times and took notes to support the interpretation of the participants’ responses. The transcriptions were translated into English to ensure that all the researchers could engage with the material, as not all were proficient in Finnish.

Following the transcription and translation, the authors prepared for the collaborative reflexive thematic analysis of the data, following Braun and Clarke [35]. The thematic analysis process consisted of six steps: familiarization with the material, finding codes, developing clusters, generating themes, examining and defining themes, and writing the report. As the process had an iterative nature, the researchers continuously moved back and forth between the different steps. Initially, each researcher individually engaged with the interview material by reading the transcripts several times. Notes were taken to support familiarization with the data. This phase was revisited throughout the analysis to ensure a deep understanding of the material. The first author identified key meaningful units, which were collectively analyzed and refined in collaboration with all the authors in a shared document and developed into codes (*n* = 68) through iterative discussions. Thereafter, the themes were developed through a collaborative process, where the authors grouped the codes into clusters (*n* = 6) by frequently revisiting earlier steps to reassess and adjust the coding. Through repeated rounds of analysis and reflection, the clusters were further refined, ultimately resulting in two main themes and six subthemes that encapsulate the essence of the material.

### 2.6. Rigor

SRQR were adhered to in this study [36]. The research process in this study has been described in detail, allowing readers to evaluate its relevance and applicability in other contexts to strengthen transparency and dependability. Additionally, a previous chapter outlines the inclusion and exclusion criteria, as well as participant characteristics, offering important context for understanding the scope of the findings.

This study adhered to the Braun and Clarke’s reflexive thematic analysis framework [35], which emphasizes researcher subjectivity and reflexivity throughout the analysis process. All the authors have a background as registered nurses, and some of the authors were experienced in nursing leadership, thus demonstrating familiarity with the study setting and context. The first author, who collected the data and carried out the interviews, had practical experience in physical homecare and had been introduced to the telehomecare service, which fostered a comfortable atmosphere during the interviews and facilitated a mutual understanding between the interviewer and the participants. Furthermore, the first author was experienced in qualitative methodologies, particularly in conducting digital interviews. To minimize bias and uphold credibility, leading questions were consciously avoided.

The data analysis was carried out by authors from diverse academic and professional backgrounds, including nursing, nursing leadership, caring sciences, and sustainability. This diversity enriched the reflexive dialogue and critical reflection that were central to the thematic analysis process. Initially, each of the five researchers conducted an independent analysis of the data. These analyses were followed by collaborative in‐depth discussions, which enhanced the confirmability and trustworthiness of the findings by fostering openness to differing interpretations and critical reflection on personal biases. During the analysis, the authors systematically reviewed the responses to all nine questions in the interview guide. To maintain analytical rigor and study focus, four questions were included. Five questions that did not generate additional insights relevant to the study aim were excluded from the final analysis.

### 2.7. Ethical Considerations

Ethical considerations were central to the study design and implementation. The study adhered to ethical research standards in accordance with the World Medical Association’s Declaration of Helsinki (1964) [41] and the guidelines issued by the Finnish National Board on Research Integrity [42]. Ethical approval was granted by the well‐being services county (approval number: 1538/13.01/2023). Informed consent was obtained from all the individuals concerned before data collection began, and the participants received comprehensive information about the study’s purpose, methodology, and data management. This information and these documents were provided through e‐mail and verbally before the interviews. All the participants were aware of the voluntary nature of the study and were assigned numerical identifiers to ensure confidentiality.

## 3. Results

**FIGURE 1:**
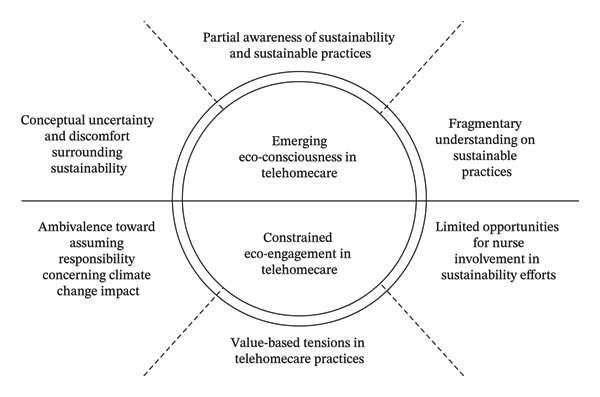
Overview of practical nurses’ perceptions of and engagement with sustainability, with the horizontal axis representing the tension between the emerging consciousness and constrained engagement.

The analysis resulted in the two main themes, *Emerging Eco-Consciousness in Telehomecare* and *Constrained Eco-Engagement in Telehomecare*, with three subthemes each (Figure 1). These themes capture the nuanced perceptions and engagement of practical nurses regarding sustainability within the telehomecare setting.

### 3.1. Emerging Eco‐Consciousness in Telehomecare

#### 3.1.1. Conceptual Uncertainty and Discomfort Surrounding Sustainability

All the participants in this study expressed discomfort when asked about sustainability and climate change impact in the context of their work. When asked what sustainability is in the nursing care context and what role practical nurses play concerning climate change, all the participants expressed some form of nonverbal response, such as sighing, staying silent, or nervously laughing, indicating uncertainty or hesitation about engaging with the concept. When asked about sustainability in nursing care practices, one participant responded as follows:

Yeah. How should I answer that? ^∗^silence^∗^ Shall we skip over this question? ^∗^laughter^∗^. The sustainability thing is… I am not sure what to answer… (P8)

Furthermore, the participants demonstrated a misconception that sustainability concerns the complete replacement of physical care with exclusively digital forms of care. When asked what sustainability means in the nursing care context, one participant articulated their perception as follows:

The thing is, the healthcare sector is never going to end. We cannot be replaced by robots. There will probably never be a robot that can do all the home visits that we need. This is a sector that will always be and will remain. Let us at least hope so. But it is a bit hard to answer that topic – sustainability and the healthcare sector. (P8)

#### 3.1.2. Partial Awareness of Sustainability and Sustainable Practices

The participants expressed that most practical nurses carry out their duties without consciously reflecting on sustainability, indicating nurses’ limited awareness of sustainable practices. Sustainability is generally not a consideration in daily telehomecare practices, as practical nurses tend to focus primarily on the client’s immediate needs.

You think about being there for the client. I actually do not think about my part in sustainable development as much… You think more from the client’s point of view, rather than what I, as a practical nurse, can do sustainably. (P3)

Many participants appeared to engage in sustainable practices, even though they did not explicitly recognize them as such. One participant noted that although everyone likely incorporates some form of sustainable practices in their work, it often goes unnoticed by the individual themselves.

I have not really thought about it. Everybody probably has some kind of sustainable development path, or an idea of it, but has just not paid attention to it. (P1)

#### 3.1.3. Fragmentary Understanding of Sustainable Practices

The participants in this study initially expressed difficulties in answering the interview questions on sustainability. However, through continued dialogue with the interviewer, all the participants were eventually able to engage with the topic. The participants identified multiple elements in their work that they considered sustainable. The majority of the participants recognized the economic aspects of sustainability in delivering nursing care at a distance, where potential savings could be achieved in terms of personnel resources and other operational costs. Several participants mentioned aspects of social sustainability, for both clients and personnel, including accessibility of care, developing stronger caring relationships, a sustainable work life, and the importance of implementing effective personnel policies. One participant discussed the social aspects of sustainability in their workplace in the following way:

It is probably first and foremost the planning. Especially if you are involved in homecare, with schedules and everything. Sustainability may also mean that people enjoy their workplace and want to stay […]. If you have a good team and all that, then you can work any way possible […]. I think that personnel policy could perhaps be a little better by listening to the carers too, about how we cope. (P10)

All the participants mentioned making use of less protective gear, fewer vehicles, and less fuel as environmentally friendly aspects, and some participants identified practices such as using a bicycle to get to work and having bins to sort trash in their office as environmentally favorable. All the participants had a strong focus on economic aspects of the service, such as that telehomecare makes service delivery more effective, decreases the costs of protective gear and car fuel, and supports physical homecare, especially during peak times of homecare visits. However, most participants were unable to reflect comprehensively on sustainability without guidance from the interviewer. Their understanding tended to focus on isolated tasks or specific aspects of their daily work, indicating a limited conceptual grasp of sustainability within the broader context of nursing care in a telehomecare context.

### 3.2. Constrained Eco‐Engagement in Telehomecare

#### 3.2.1. Ambivalence Toward Assuming Responsibility Concerning Climate Change Impact

The participants expressed the need for individuals to take responsibility for climate impact. However, several expressed uncertainty about what sustainable work practices entail and highlighted the difficulty of integrating such practices into their routines. Responsibility for sustainability was often perceived as belonging to someone else, such as individuals with more expertise or better knowledge of guidelines. Several participants highlighted that working sustainably is a collective responsibility, which one participant expressed as follows:

I think it is everyone’s responsibility to try their best at least. It does not only concern healthcare workers, but everyone on the planet. (P7)

Additionally, some of the participants described sustainability and climate change as relatively new and modern concepts, which may reflect an underlying lack of motivation to take responsibility for these issues within their professional practice. One discussed the situation as follows:

It has been a long time since I graduated as a practical nurse. I do not think this was discussed at that time. This is more of a modern‐day issue. (P6)

From an organizational and leadership perspective, the majority of the participants implied that there is a lack of discussion surrounding sustainability throughout all personnel groups. Furthermore, the participants expressed a need for more training in sustainable practices in a way that does not place the responsibility on the individual. According to some of the participants, the responsibility for sustainability training is placed on the individual nurse, which poses challenges given the time‐sensitive and demanding nature of their work environment.

Maybe they have had a lecture or something on sustainability. But not everyone pays attention to it or even checks what kind of training programs or lectures there are. So that is another thing, you have to check yourself to see what they have organized. (P7)

#### 3.2.2. Value‐Based Tensions in Telehomecare Practices

The participants deliberated on the need for active choices concerning sustainability in nursing practice to decrease the climate impact of healthcare, such as minimizing waste, greenhouse gas emissions, and energy consumption. The participants recognized that telehomecare offers opportunities for more economically, socially, and environmentally sustainable practices. However, the participants expressed that some nursing practices require unsustainable methods, which create value‐based tensions. The participants highlighted that digital forms of care, such as telehomecare, are not suitable for everyone and might even create distress for the client. One participant described the following situation:

Of course, there are clients who think they are being watched through the device […]. A client once put a piece of cardboard in front of their tablet so that we could not see them. (P5)

Furthermore, all the participants recognized telehomecare as a complement to physical homecare services. However, the participants recognized the risk of clients not receiving the care they needed. As one participant expressed:

The digital services do not clean up after you have made a mess or things like that. Or see that you actually need to change your clothes, whether you have washed yourself and taken care of your hygiene, and the follow‐up. So they [the clients] do not suffer, and they get enough food and such, it has to be both of these visits, both physical and digital, so that you can make sure. (P10)

Additionally, the participants acknowledged that telehomecare had environmental implications. Delivering care at a distance requires the use of technology, which contributes to the generation of e‐waste. Moreover, telehomecare increases electricity consumption, which can be considered environmentally challenging and introduces a financial burden on clients. One participant described it as follows:

Yes, it runs on the client’s own power, or electricity consumption, but I do not know how much it uses […]. But they do not have to pay for anything for the tablet itself or the SIM‐card. But they do have to pay for electricity, of course. (P9)

#### 3.2.3. Limited Opportunities for Nurse Involvement in Sustainability Efforts

From a leadership perspective, the participants reported limited communication and nurse involvement in sustainability efforts throughout the organization. Several participants described a lack of inclusion in sustainability development processes and policies. One participant suggested the need for a dedicated full‐time role related to sustainability within the organization, rather than assigning their tasks to someone already holding another position. Some participants were unaware of whether a designated person responsible for sustainability existed in the organization or whether sustainability issues were discussed among leaders. One participant expressed the following:

It is not something that I think we have discussed or that has been brought up generally for discussion. I think there may be such discussions among our leaders, but nothing comes “down” to us. (P3)

The participants expressed interest in becoming more engaged with sustainability efforts, although some expressed that these issues did not reach them at their level. The practical nurses also articulated a willingness to contribute to addressing climate change. When asked about their roles as care professionals in considering sustainability and the healthcare sector’s climate footprint, one answered as follows:

Yeah, I wish I could say yes. That I have a role. But I do not think… Personally, it is not something I reflect on that much, actually. (P3)

## 4. Discussion

The findings in this study showed two main themes: *Emerging Eco-Consciousness in Telehomecare* and *Constrained Eco-Engagement in Telehomecare*. The study shows that practical nurses working in telehomecare are becoming more aware of issues concerning sustainability while simultaneously experiencing dilemmas and limited influence when it comes to putting their environmental commitment into practice. To ensure comprehensive change in the nursing environment, the connection between practical nurses having eco‐consciousness and eco‐engagement is crucial, that is, working sustainably must become both a wholehearted commitment and an active choice for the practical nurse as an individual [4, 17]. Below, we discuss the findings and how leadership can foster both eco‐consciousness and eco‐engagement, drawing on previous research and theoretical frameworks.

### 4.1. Leadership Fostering Eco‐Consciousness in Telehomecare Nursing

The findings in this study indicate that practical nurses lack a holistic understanding and knowledge of sustainability, including its social, environmental, and economic aspects [2, 3], aligning with previous research [8, 18, 23]. The participants experienced difficulties and discomfort in responding to questions on sustainability, indicating a limited eco‐consciousness among telehomecare nurses. However, through dialogue with the interviewer, the participants were able to identify numerous aspects of their work that can be considered sustainable. This finding suggests that while practical nurses may possess fragmented knowledge, the absence of a comprehensive understanding limits their ability to recognize how their practices either contribute to or mitigate climate impacts. Professionals within healthcare settings need more knowledge to understand the relevance of sustainability within their line of work [8], especially in a digital context. However, the participants in this study expressed a form of silent knowledge about sustainability, where practical nurses unknowingly perform sustainable actions in their daily routines, possibly implying a lack of conceptual awareness or confidence in their abilities to engage meaningfully with sustainability in their professional role. This finding might also suggest the absence of a shared culture of sustainability within telehomecare and within the organization more broadly, where sustainable practices are normalized and placed within existing routines. Such an organizational culture is fundamental to the implementation of new policies [11, 43] and could be strengthened by creating forums for peer‐to‐peer knowledge exchange [43].

Nursing leaders play a key role in fostering this culture of change by supporting [10, 15], motivating, and engaging change [13]. Additionally, nursing leaders need to make sure healthcare personnel have the level of competence needed [16] to integrate sustainability into their professional practice. However, leaders’ ability to guide practical nurses in adopting sustainable working practices is limited by their own level of knowledge and climate–health literacy [10], indicating that leaders may require further training and development in this domain. Green leadership training might enhance leaders’ competencies and thereby strengthen the implementation of sustainable practices [43]. According to Pinzone et al., sustainability training can also have positive impacts on job satisfaction [44], which aligns with sustainability goals.

This study was conducted within the telehomecare context, thereby extending previous research—which has predominantly examined sustainability in nondigital settings [30, 31]—by highlighting the research gap in digital care environments. While the digitalization of healthcare services can reduce the sector’s overall climate impact [6, 7], it might simultaneously introduce additional burdens and generate new challenges in delivering healthcare services [45]. More research is needed to determine which patient groups and health conditions can appropriately utilize digital services in ways that are socially, financially, and environmentally sustainable. Furthermore, the participants expressed the misconception that sustainable healthcare means all physical care being replaced with digital care, highlighting the critical need for further education and training in sustainability. These findings suggest a broader need for comprehensive education in sustainable healthcare practices, in line with Stanford et al., suggesting the implementation of quality improvement and environmental sustainability within healthcare education [46]. Educational efforts are particularly necessary in digital care settings, where sustainability practices are less established than in traditional, physical care environments. The aspect of healthcare professionals’ competences is also central in the operational framework by the World Health Organization [47], as well as the policy report by the World Health Organization on green skills for a sustainable future [24], where healthcare professionals’ green skills and climate–health literacy are pinpointed as essential in advancing more climate‐resilient healthcare systems. Educational initiatives are fundamental to advancing environmentally sustainable healthcare systems by informing institutional strategies and guiding policy development [27].

### 4.2. Leadership Mobilizing Eco‐Engagement in Telehomecare Nursing

The findings in this study indicate that practical nurses are somewhat ambivalent toward taking responsibility in relation to the climate impact of healthcare and implementing more sustainable everyday practices. This finding might imply that practical nurses are overburdened and have to prioritize patient work [8, 19]. This may impact their possibilities and motivation to engage with the subject. Some of the participants distanced themselves from assuming responsibility, potentially due to the lack of agency [48] and a negative attitude toward change, as previously reported by Thomas and Suresh [49]. Additionally, a sense of conditional accountability emerged whereby telehomecare nurses’ willingness to engage in sustainable practices was connected to the expectation that others would do the same. As being eco‐engaged is an individual choice [17], this study highlights the importance of a transformational leadership style that can motivate change among practical nurses [13] and cultivate a green work environment with green intentions, behaviors, and creativity [50]. According to Li et al., ethical leadership styles have been proven to engage nurses in more sustainable behaviors [51]. Furthermore, the participants in this study navigated the tension between the importance of sustainability and the imperatives of patient care and nursing practice, such as choosing clients suitable for telehomecare. This dynamic creates conditions for value‐based tensions in telehomecare. Delivering care at a distance contributes to the generation of e‐waste and increases clients’ electricity consumption, which raises environmental sustainability concerns and potential financial burdens for clients. The participants highlighted that telehomecare may not be appropriate for all client groups, raising concerns about an increased risk of missed or inadequate care. Such gaps in care delivery can have financial implications for both service users and healthcare providers, as unmet needs may have serious clinical consequences and lead to increased long‐term costs [52]. This conflict in professional values might impact the level of engagement of practical nurses. Therefore, it is essential for leadership to involve healthcare personnel at an early stage in organizational change processes to mitigate emerging resistance and identify potential challenges [14].

Furthermore, the participants in this study expressed limited opportunities for nurse involvement in sustainability efforts. Many participants assumed that their leaders were engaged in discussions on sustainability and expressed a lack of communication between “higher‐ups” and practical nurses. According to Baykara Mat and Multu, some organizations might see sustainability as a marketing strategy to uphold an image of environmental responsibility rather than a genuine commitment to sustain the environment [14]. Several participants in this study indicated a desire for greater involvement in sustainability‐related initiatives. Opportunities for involvement, such as organizing task forces or appointing champions, have been shown to positively influence personnel engagement [43]. The combination of limited participant involvement and a range of external constraints, including weak policy frameworks and inadequate infrastructure [8, 22], competing priorities and resource scarcity [19], insufficient organizational support and commitment [9, 20, 22, 26], and hierarchical barriers to participation [9, 22], highlights the urgent need for ethically grounded, transformative leadership. Furthermore, this raises the question of whether the lack of opportunities and structural conditions to influence decisions and work conditions, as well as practical nurses’ perceived inadequate preparation for handling repercussions of climate change, may contribute to their experiences of ethical stress, given that sustainability in healthcare can be considered ethically important [4, 8].

This study extends existing sustainability research in nursing by demonstrating how sustainability is negotiated in a digital care context, where eco‐consciousness does not automatically translate into eco‐engagement, and where leadership and organizational conditions play a critical role in shaping this gap. Healthcare organizations bear the responsibility for establishing the necessary conditions, such as structured opportunities for discussion and training initiatives, for healthcare personnel to engage with sustainability‐related practices and policies. Collaborative efforts are emphasized as key to sustainable transformations [8, 9, 22] to meaningfully engage with sustainability within the context of digital nursing care. Such efforts could foster collaborative decision‐making and drive systemic change toward environmentally responsible practices. The participants expressed that the adoption of new behaviors requires considerable effort, a finding consistent with previous research [53]. Consequently, such initiatives should be prioritized, and appropriate resources should be allocated to support these efforts, including designated time for sustainability‐related training. Moreover, political enablers are widely regarded as critical for advancing climate policy agendas [54]. However, the absence of robust empirical data and comprehensive policy frameworks for achieving and sustaining environmentally sustainable healthcare systems is underscored [27].

### 4.3. Limitations

This study was conducted in Finland and involved participants from a single well‐being services county, which may be considered a limitation. Since homecare services, healthcare systems, and their delivery differ across countries, the generalizability of the results might be restricted. Another limitation is the lack of gender diversity, as all the participants were female; no suitable male participants were identified for inclusion. A more gender‐diverse sample might have further strengthened the transferability of the findings, as previous studies have demonstrated gender‐related differences in environmental behavior and environmental understanding [55]. On the other hand, the participants represented diverse backgrounds and differed in the amount of experience they had with the telehomecare service. While a few were highly familiar with the service and had worked with it for up to 7 years, the majority had less than 1 year of experience. A similar pattern was observed in the participants’ nursing experience, where a notable variation existed despite a relatively high median. This variation influences the transferability of the findings, as it may have affected how participants perceived sustainability and discussed nursing activities in relation to sustainability. Although the sample size was relatively small, 10, it was considered adequate because sufficient information power was obtained [40].

This study chose to use individual interviews, as the subject of sustainability can be considered politically charged and, therefore, sensitive. The participants were given the option to be interviewed face‐to‐face or digitally, and they all requested digital interviews. Since all the participants operated within a digital work environment, conducting interviews in this format aligned well with the principle of studying individuals in their natural setting. However, even though digital interviews can encourage more thoughtful responses and eliminate the need for travel, they can limit the observation of nonverbal communication and may affect the researcher’s ability to foster a positive interview atmosphere [56]. Furthermore, the first author’s previous experience in conducting digital interviews contributed positively to the study’s reliability.

Before the interviews, the participants were introduced to the concept of sustainability [2, 3], which may have influenced their responses. The researcher conducting and transcribing the interviews had clinical experience as a practical nurse and registered nurse in the field of physical homecare and at‐home hospital care. This background served as a strength of the study, providing the researcher with a comprehensive understanding of nursing duties and working conditions. However, none of the authors had work experience with the telehomecare services. The study included four out of the nine interview questions developed for the interview guide. While this decision may have limited the breadth of sustainability‐related perspectives, it did not constrain the depth of analysis within the applied analytical framework. Furthermore, it is important to acknowledge that thematic analysis is inherently subjective. However, rather than attempting to eliminate researcher influence, the researchers actively reflected on it throughout the analysis, recognizing it as a vital component of reflexivity and a source of insight in the interpretive process [35].

## 5. Conclusions

In this study, practical nurses’ perceptions of and engagement with sustainability within telehomecare were explored. The findings show a need to balance practical nurses’ eco‐involvement and the contextual constraints in the digital caring context. Moreover, the results reveal the need to strengthen practical nurses’ eco‐consciousness and eco‐engagement in work to create a resilient organization with a workforce prepared for the challenges brought on by climate change.

The findings of this study underscore the need to enhance healthcare professionals’ and leaders’ consciousness and awareness of the implications of climate change and the importance of sustainability within the healthcare sector. The findings suggest that more training and education in sustainability in healthcare are needed to strengthen engagement. Such initiatives can strengthen motivation to adopt eco‐conscious approaches, empower leadership to advocate for a greener, sustainable transformation, and promote structural development within organizations toward greener operations. These efforts require dedicated financial resources to ensure their implementation and continuity. The study further highlights the importance of transformative and ethically grounded leadership that champions green innovation and fosters a culture of sustainability and interprofessional collaboration. To enable the successful integration of environmentally sustainable practices into healthcare delivery, robust frameworks are needed to support the development of a resilient and future‐oriented welfare system.

## Author Contributions

Conceptualization and study design: M.R., A.A., L.E., M.A., and L.F.

Data collection and visualization: M.R.

Analysis and interpretation: M.R., A.A., L.E., M.A., and L.F.

Manuscript drafting and critical revision: M.R., A.A., L.E., M.A., and L.F.

All authors have contributed significantly to the work and agree to be held accountable for its content.

## Funding

The corresponding author (M.R.) received a work grant from Åbo Akademi (grant number: 179/2023). The funding source had no influence on the study design, data collection, data analysis, or conclusions. No other grants from any funding agency in the public, commercial, or not‐for‐profit sectors were received.

## Disclosure

All authors agreed to the final version of the manuscript.

## Conflicts of Interest

The authors declare no conflicts of interest.

## Data Availability

The data that support the findings of this study are available from the corresponding author upon reasonable request.
